# Supplementary figures and images for: The RNA binding protein DND1 is elevated in a subpopulation of pro-spermatogonia and targets chromatin modifiers and translational machinery during late gestation
Source: PLoS Genet. 2023 Mar 1;19(3):e1010656. doi: 10.1371/journal.pgen.1010656 (PMC10010562; doi:10.1371/journal.pgen.1010656)

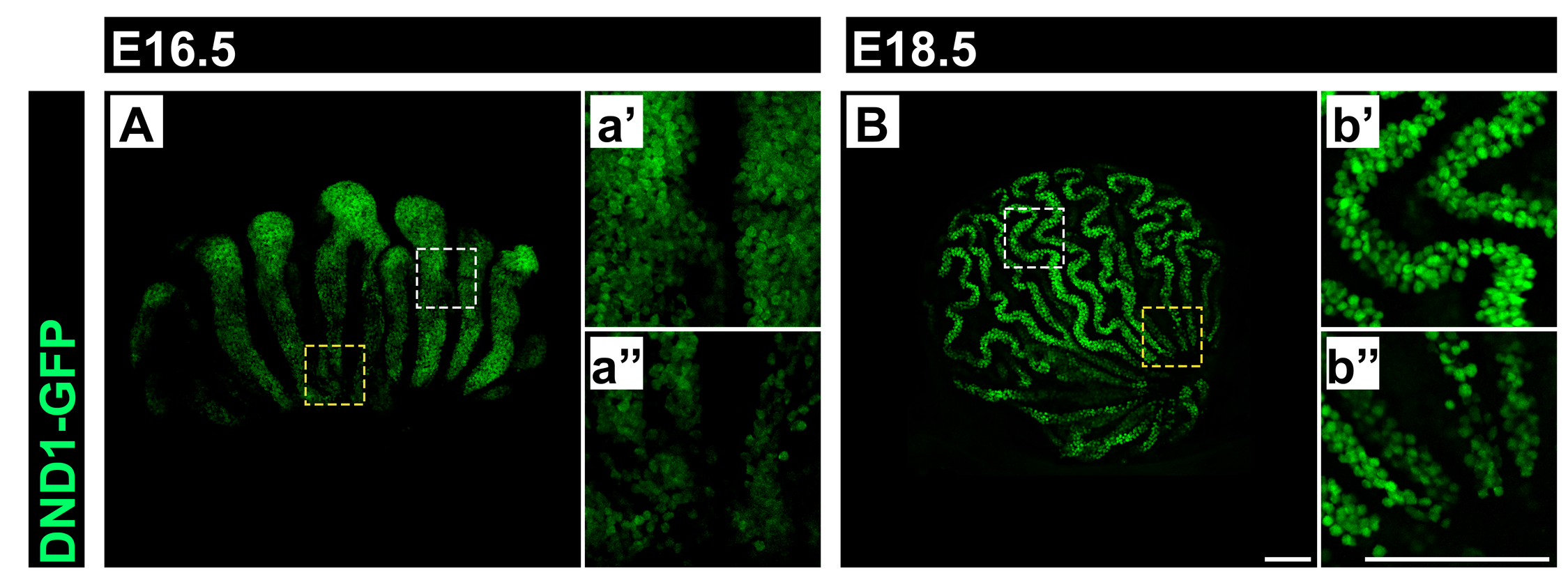

Supplement: S1 Fig — Whole mount confocal imaging of unstained freshly dissected A: E16.5 and B: E18.5 DND1-GFP testes showing heterogenous endogenous DND1-GFP fluorescence. Insets show cord region (white dotted box, a’ and b’) and rete testis region (yellow dotted box, a” and b”). All scale bars are 200μm. Scale for A and B in B, scale for a’, a”, b’, and b” in b”. (TIF) [file pgen.1010656.s006.tif]

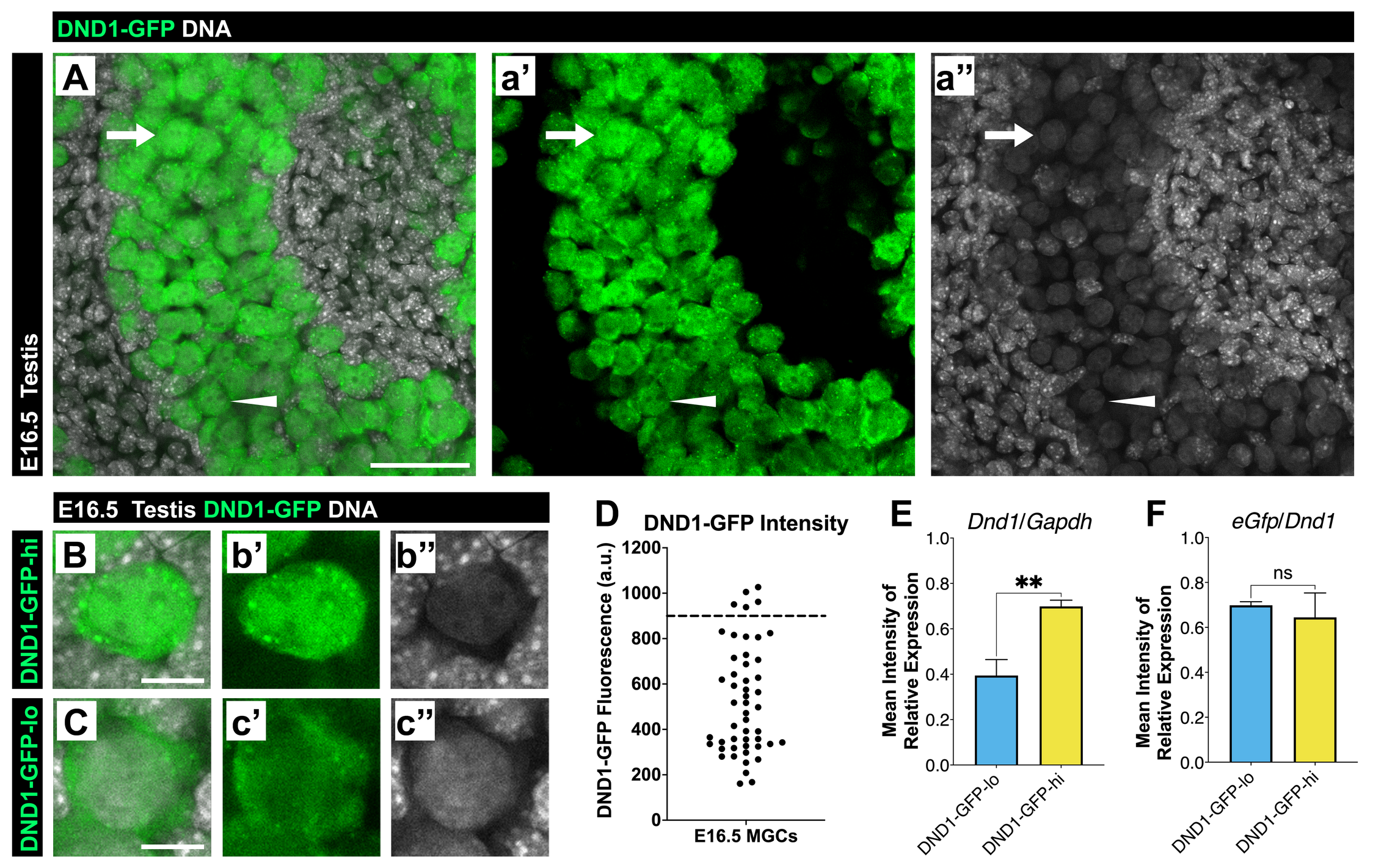

Supplement: S2 Fig — A: View of fixed MGCs in a testis cord with DND1-GFP-hi cell marked by an arrow and a DND1-GFP-lo cell marked by a wedge. Scale bar = 50μm. B, C: Representative images showing a quantified DND1-GFP-hi cell (B) and a quantified DND1-GFP-lo cell (C) from an E16.5 testis. b’, c’: DND1-GFP (endogenous signal). b”, c”: DNA (Hoechst stain). Scale bar = 10μm. D: Quantification of DND1-GFP fluorescence in 50 randomly selected DND1-GFP MGCs from multiple testes cords in 2 separate mice. Dashed line demarcates the relative fluorescence boundary between DND1-GFP-lo cells and DND1-GFP-hi cells (below and above the line, respectively). E: Mean intensity of Dnd1 expression relative to Gapdh expression in DND1-GFP-lo (blue) and DND1-GFP-hi (yellow) cells from Dnd1GFP/+ heterozygotes. F: Mean intensity of eGfp expression relative to Dnd1 expression in DND1-GFP-lo and DND1-GFP-hi cells from Dnd1GFP/+ heterozygotes. E-F: Technical replicates = 4, biological replicates ≥ 4 fetal testes. P value: ** < 0.01, n.s. = not significant. (TIF) [file pgen.1010656.s007.tif]

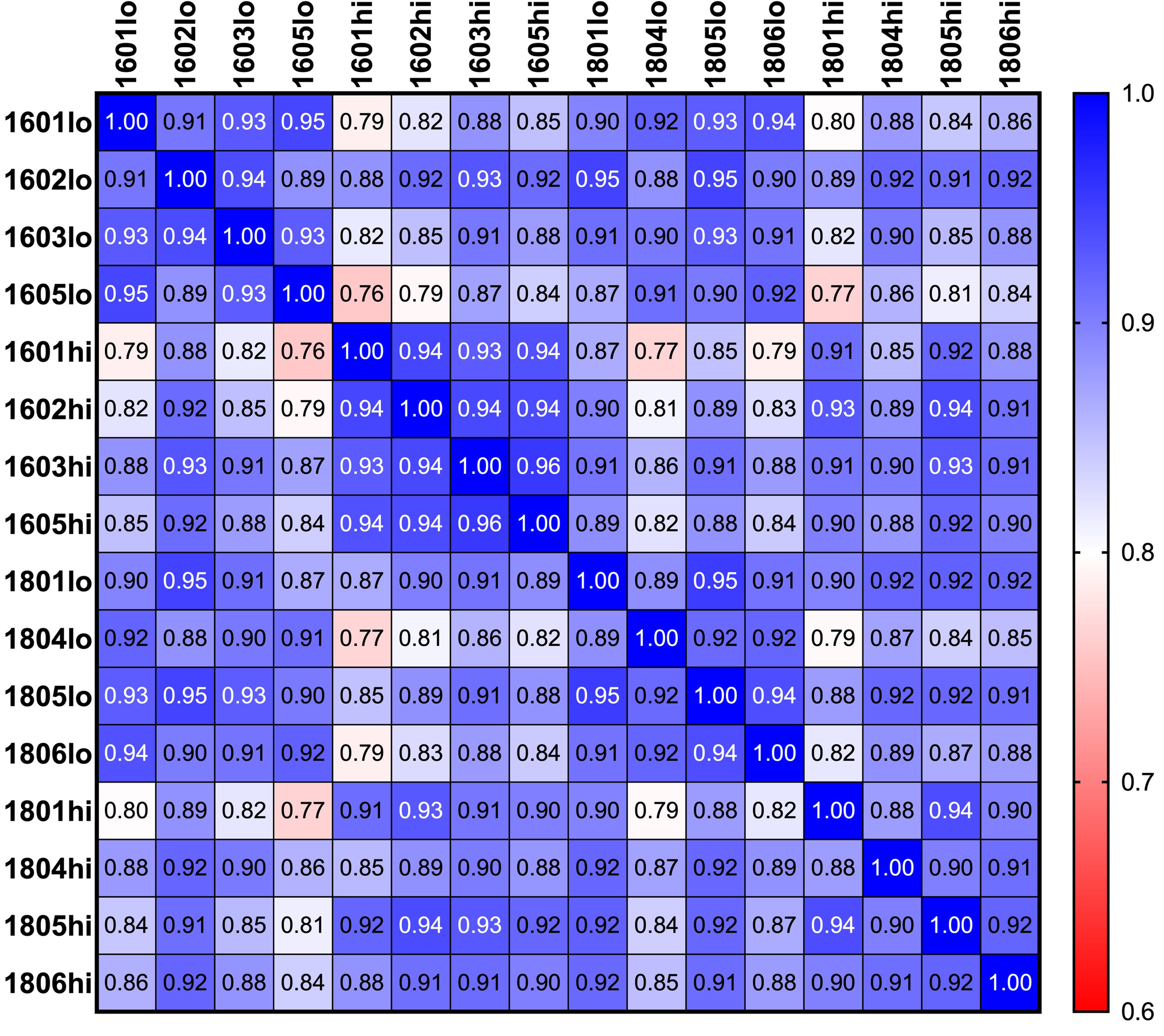

Supplement: S3 Fig — The matrix shows that DND1-GFP-lo and DND1-GFP-hi cells are most similar to biological replicate samples, but all samples of male germ cells share high similarity. (TIF) [file pgen.1010656.s008.tif]

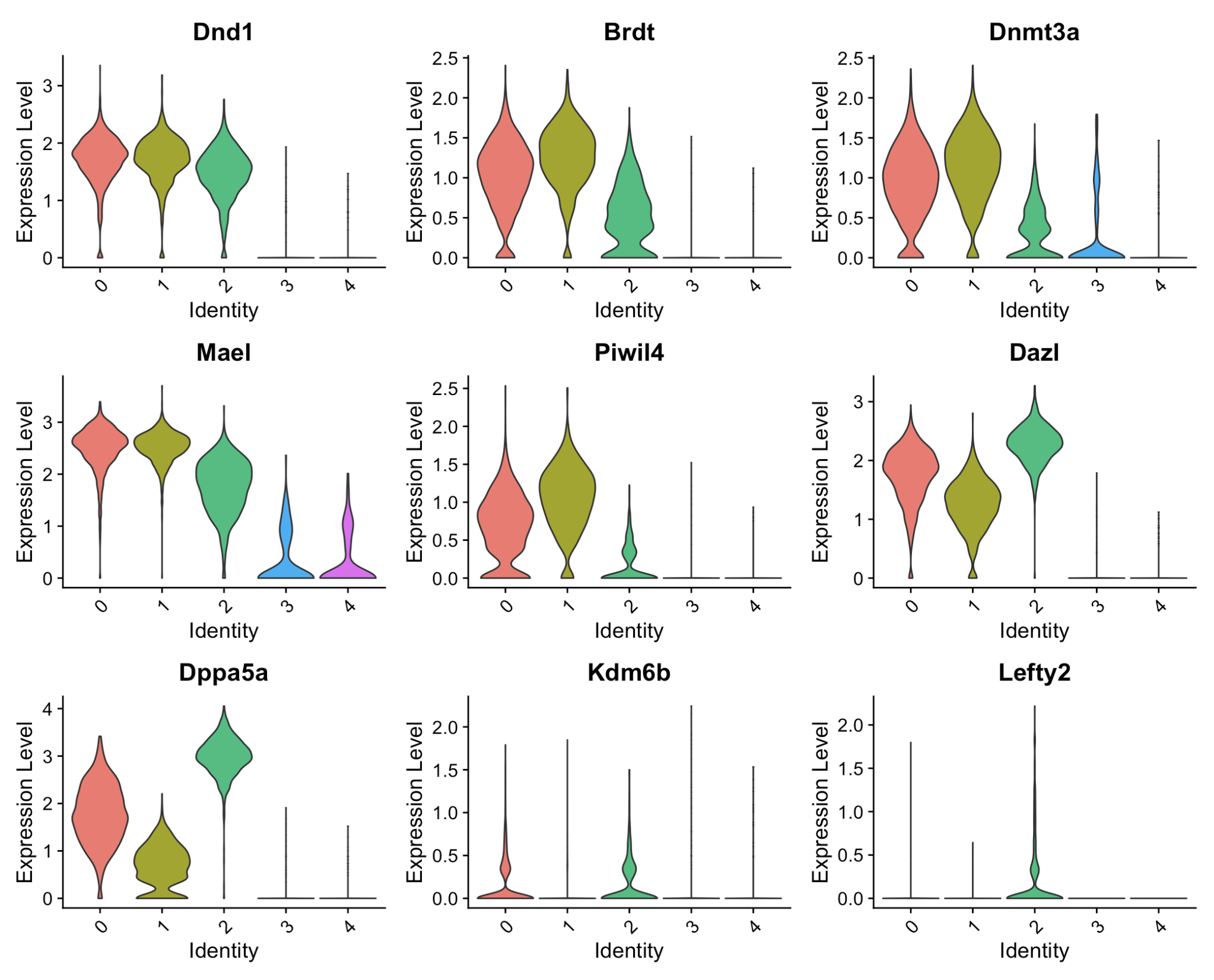

Supplement: S4 Fig — Re-analysis of E16.5 male germ cell scRNA-seq from [31]. Cluster 0 and Cluster 1 have higher levels of Dnd1 and Cluster 2 has lower levels of Dnd1 (also Fig 1E). Violin plots showing levels of other germ cell genes differentially expressed between DND1-GFP-lo and DND1-GFP-lo cells in scRNA-seq of E16.5 male germ cell from [31]. (TIF) [file pgen.1010656.s009.tif]

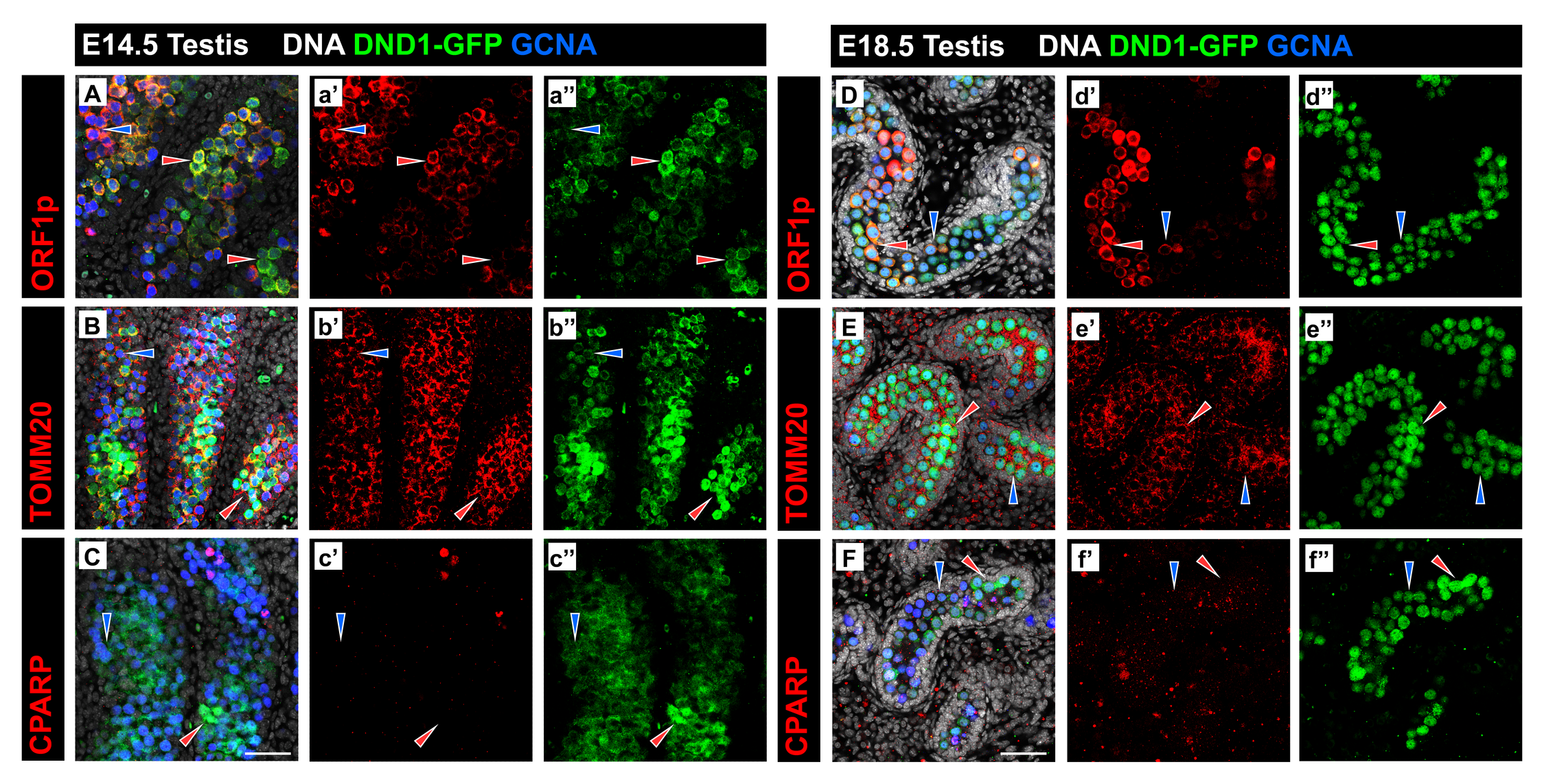

Supplement: S5 Fig — Imaging from whole mount confocal microscopy on E14.5 DND1-GFP testes (A-C) and E18.5 DND1-GFP testes (D-F) for: ORF1p (A & D), TOMM20 (B & E), and CPARP (C & F) (red). All sections stained for DND1-GFP (GFP, green), germ cell nuclear antigen (GCNA, blue), and DNA (Hoechst, white). Blue triangles mark DND1-GFP-lo and red triangles mark DND1-GFP-hi germ cells. Scales in C and F are 50μm and apply to all images. (TIF) [file pgen.1010656.s010.tif]

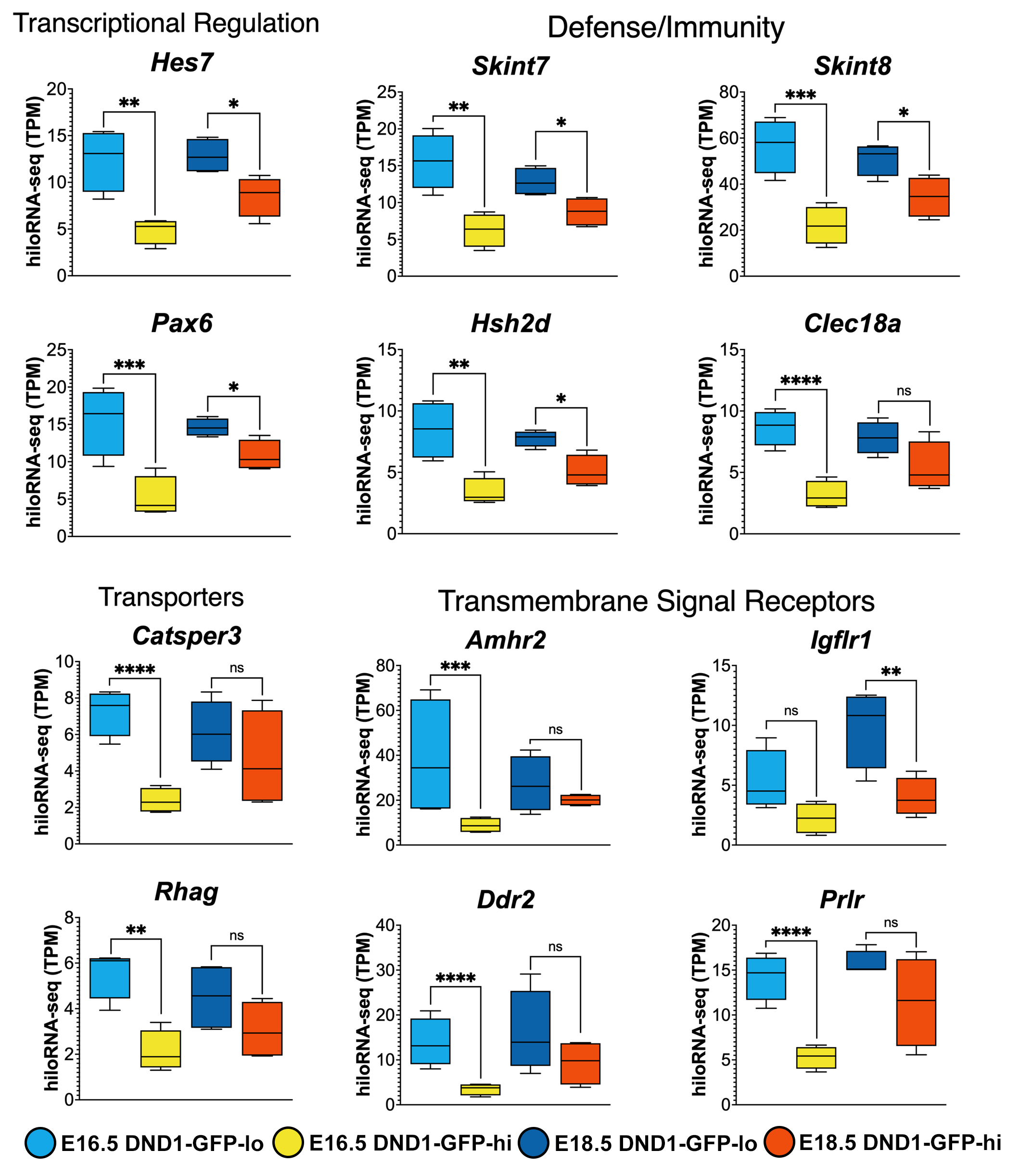

Supplement: S6 Fig — hiloRNA-seq expression P-value (DESeq2) between DND1-GFP-lo and DND1-GFP-hi cells at E16.5 and E18.5: not significant (ns), <0.05 (*), <0.01 (**), <0.001 (***), <0.0001 (****). (TIF) [file pgen.1010656.s011.tif]

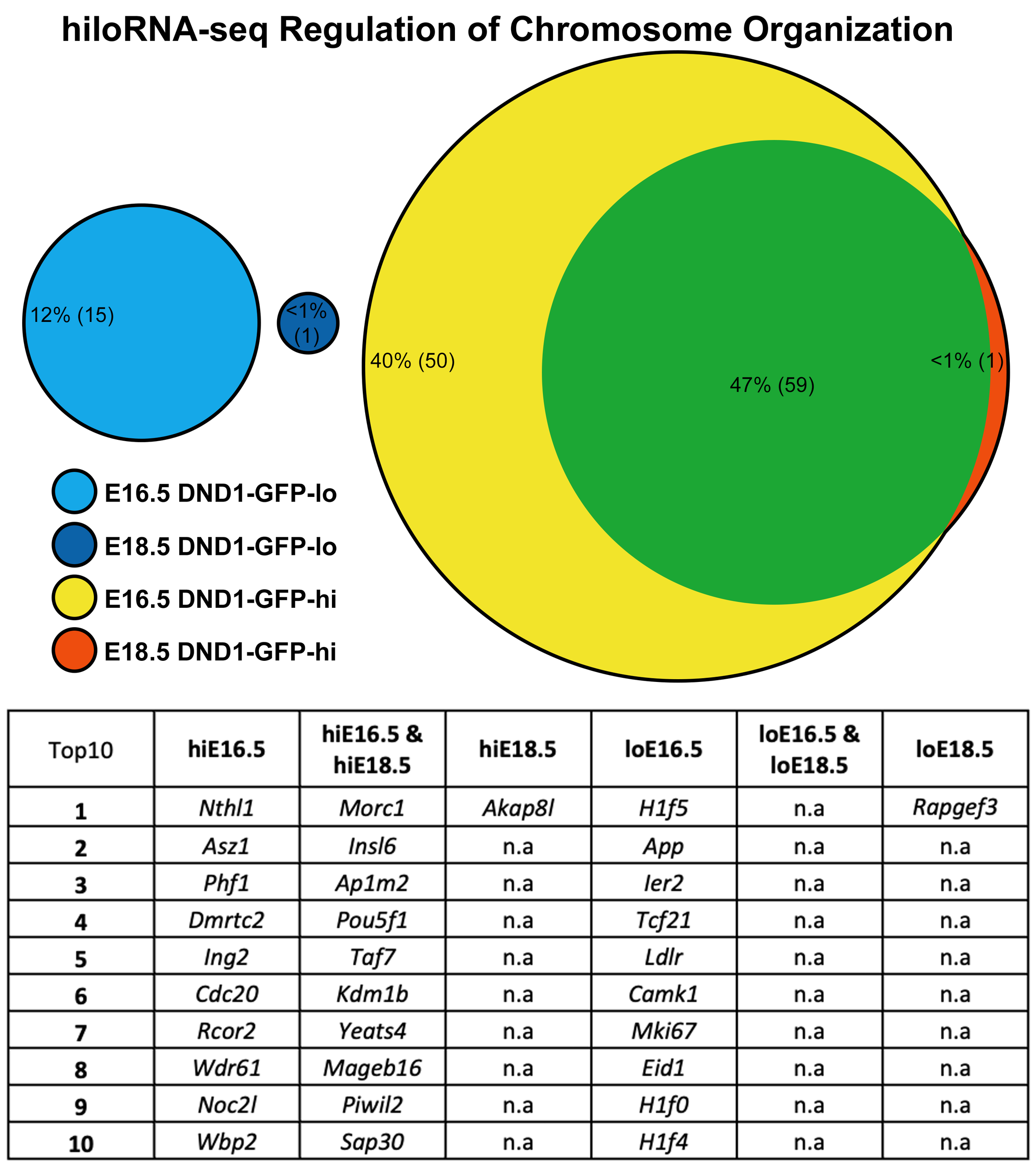

Supplement: S7 Fig — Euler plot of significant differentially expressed genes that are part of the GO biological process category “regulation of chromatin modification”. There are no genes differentially expressed in both E16.5 and E18.5 DND1-GFP-lo cells. Chart shows top 10 genes for each region of the Euler plot (for full list see S2 Table). (TIF) [file pgen.1010656.s012.tif]

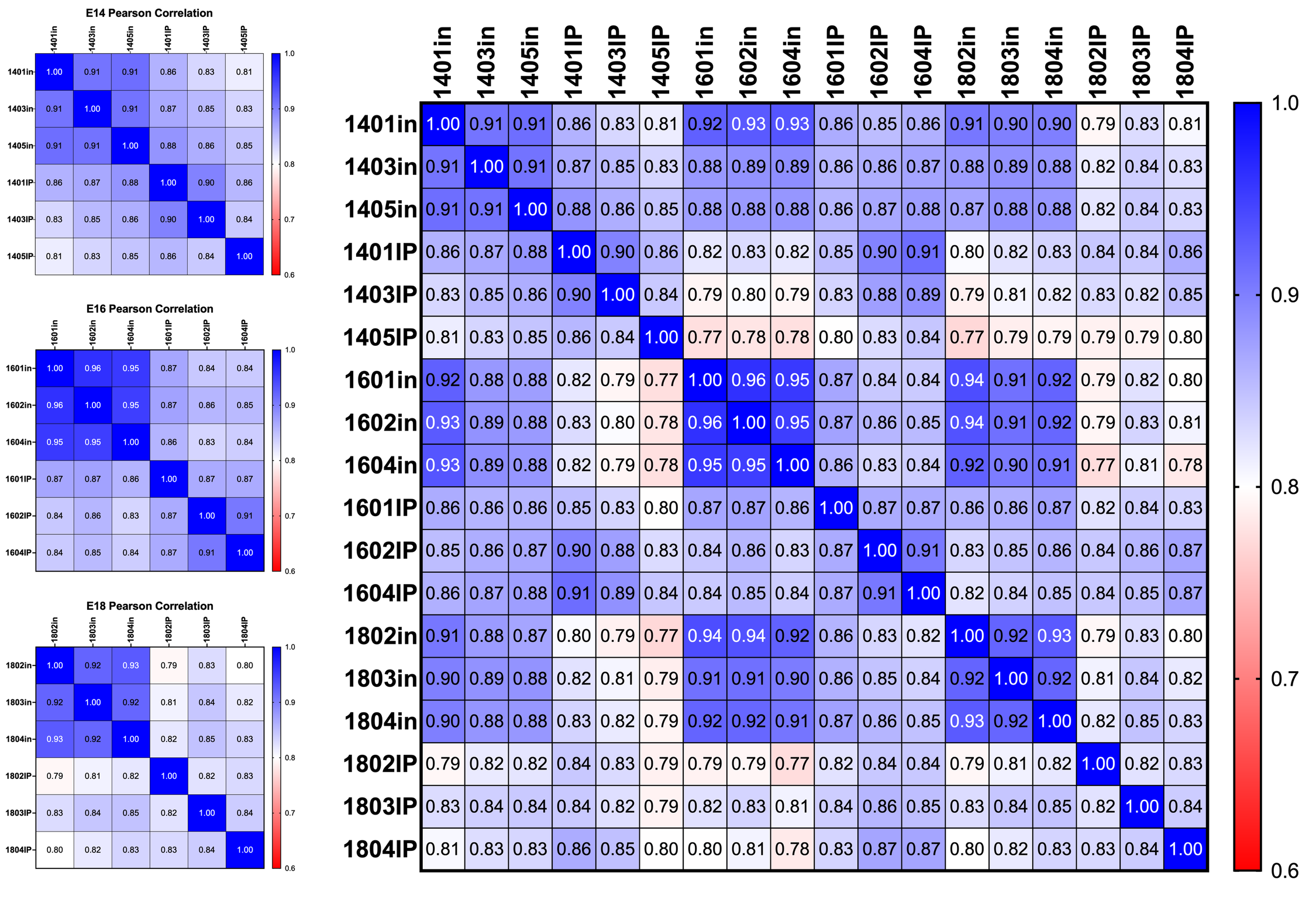

Supplement: S8 Fig — Pearson correlation matrix of input and IP biological replicates at E14.5, E16.5, E18.5 used in RIP-seq with breakouts showing isolated correlations for input and IP biological replicates at each time point. (TIF) [file pgen.1010656.s013.tif]

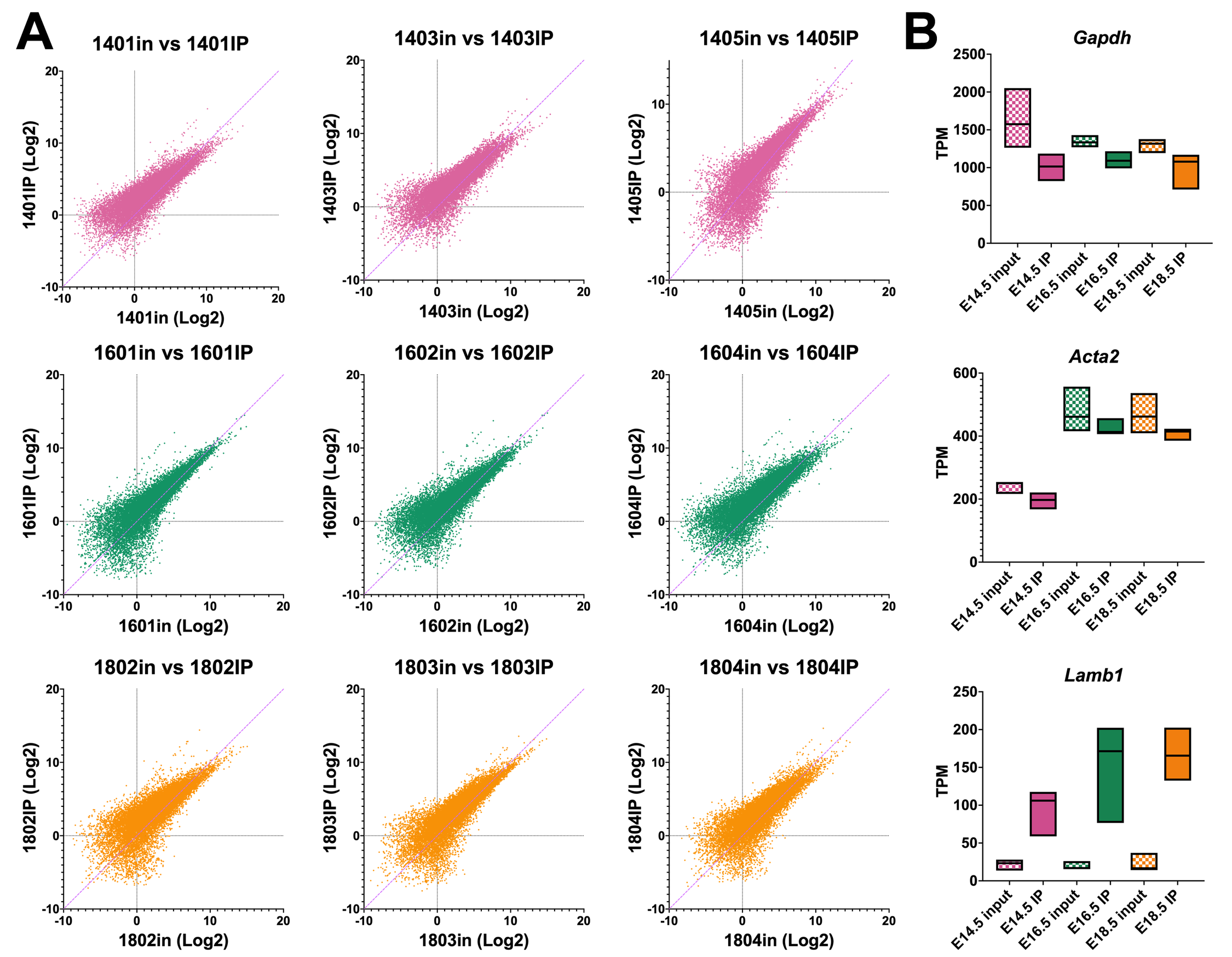

Supplement: S9 Fig — A: Scatter plots of log2(TPM) for paired input and immunoprecipitation (IP) biological replicates at E14.5, E16.5, E18.5. Guideline with slope of 1 is plotted to help delineate genes in quadrant I that are above the line, indicative of being a transcript target of DND1. B: Gene expression values (TPM) for input and IP biological replicates at E14.5, E16.5, E18.5 for highly expressed housekeeping genes that did not enrich as targets (Gapdh, Acta2), and a highly expressed gene that did enrich as a target (Lamb1). (TIF) [file pgen.1010656.s014.tif]

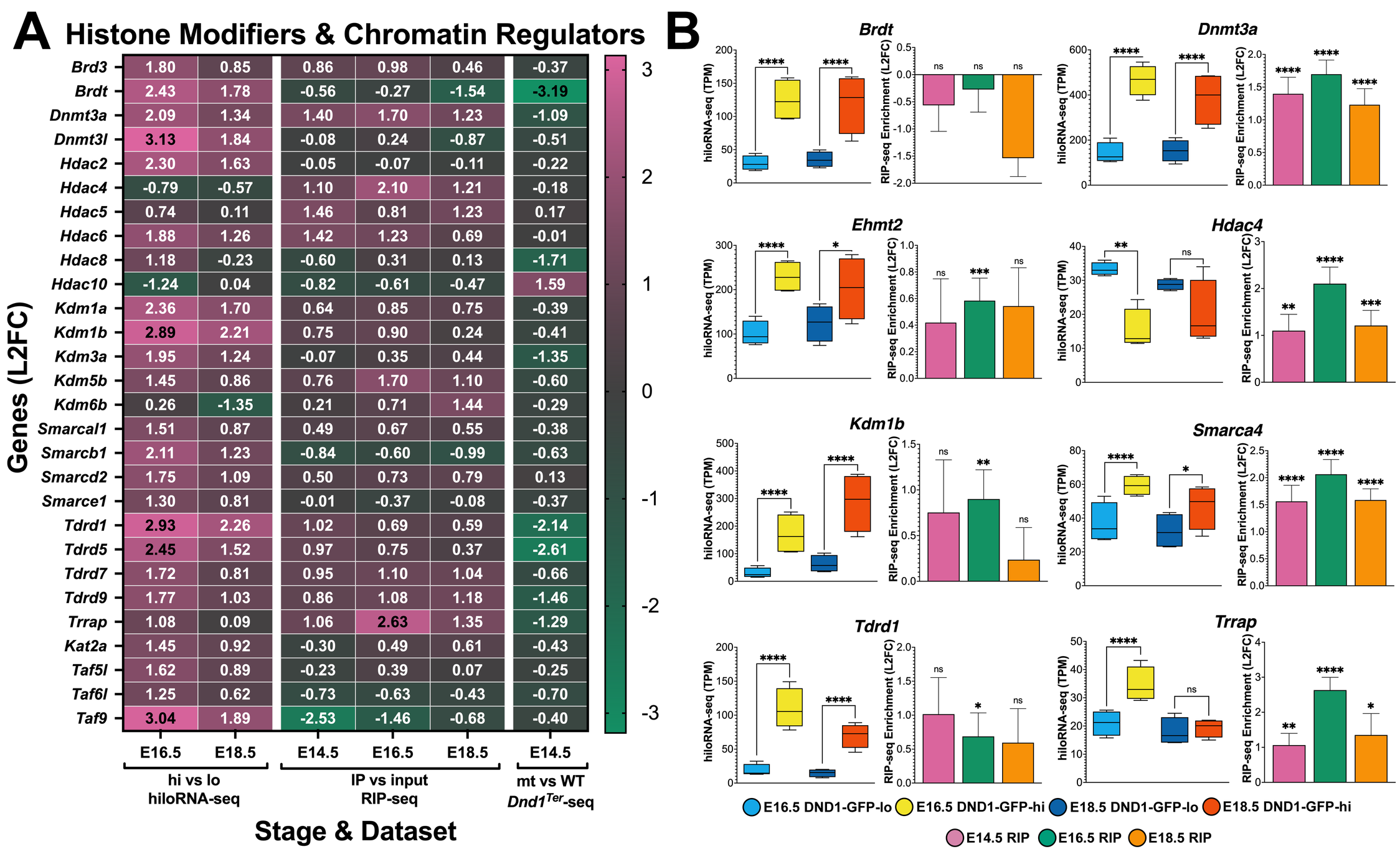

Supplement: S10 Fig — A: Heat map of epigenetic regulators showing: L2FC in DND1-GFP-hi vs DND1-GFP-lo cells from hiloRNA-seq (E16.5, E18.5); enrichment of transcript as a DND1 target as a function of L2FC in RIP-seq (E14.5, E16.5, E18.5); and L2FC in Dnd1Ter/Ter mutant vs wild type germ cells from Dnd1Ter-seq (E14.5) from [23]. B: Expanded gene-level hiloRNA-seq and RIP-seq data for representatives from each epigenetic regulator family in Figs 4B and S10A. With the exception of Hdac4, transcript levels for all genes shown are higher in DND1-GFP-hi cells, and all genes except Brdt map as significant targets of DND1 at one or more stages. hiloRNA-seq expression P-value (DESeq2) between DND1-GFP-lo and DND1-GFP-hi cells at E16.5 and E18.5 and RIP-seq enrichment P-value (DESeq2): not significant (ns), <0.05 (*), <0.01 (**), <0.001 (***), <0.0001 (****). (TIF) [file pgen.1010656.s015.tif]

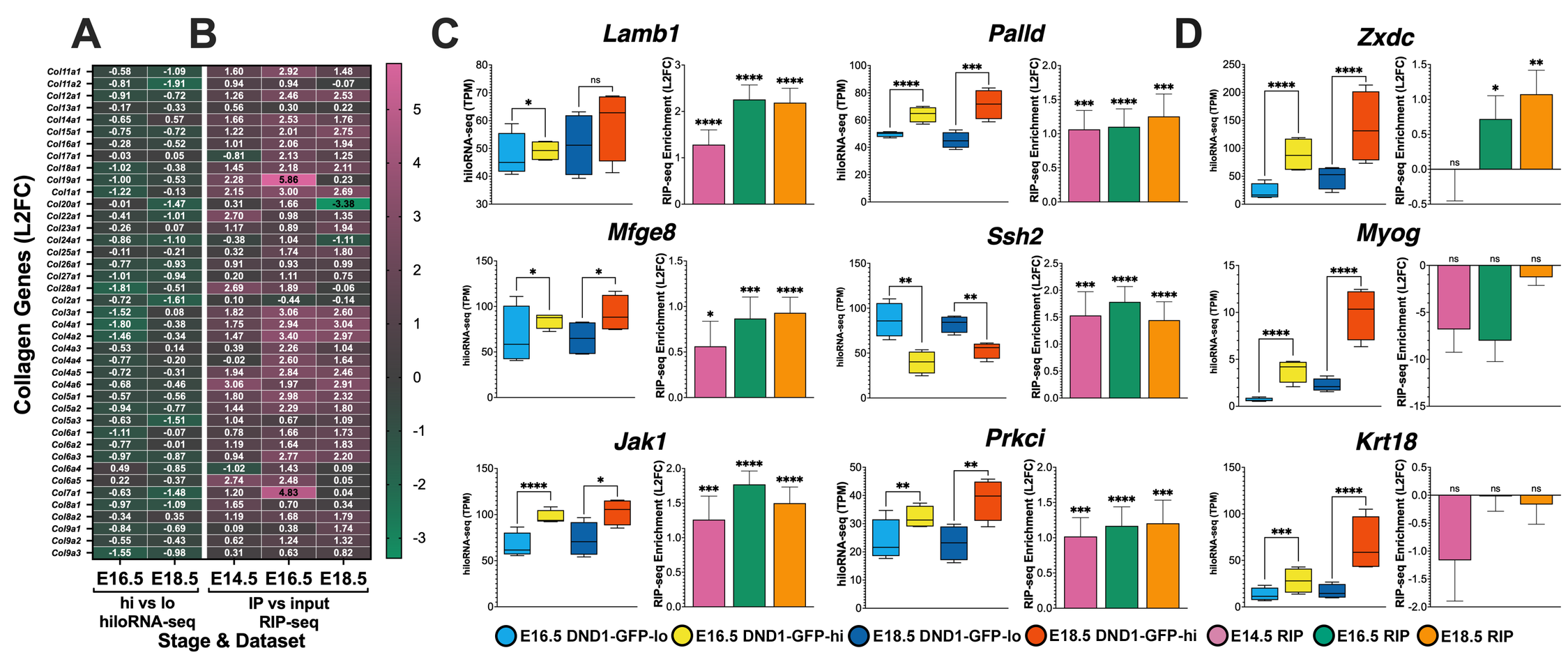

Supplement: S11 Fig — A: Heat map of collagens showing L2FC in DND1-GFP-lo vs DND1-GFP-hi cells from hiloRNA-seq (E16.5, E18.5). B: Enrichment of transcript as DND1 targets as a function of L2FC in RIP-seq (E14.5, E16.5, E18.5). C, D: Expanded gene-level hiloRNA-seq and RIP-seq data for representative genes associated with (C) intermediate pro-spermatogonia identity as reported in [55], and (D) pro-spermatogonia to spermatogonial stem cell transition as reported in [16]. hiloRNA-seq expression P-value (DESeq2) between DND1-GFP-lo and DND1-GFP-hi cells at E16.5 and E18.5 and RIP-seq enrichment P-value (DESeq2): not significant (ns), <0.05 (*), <0.01 (**), <0.001 (***), <0.0001 (****). (TIF) [file pgen.1010656.s016.tif]

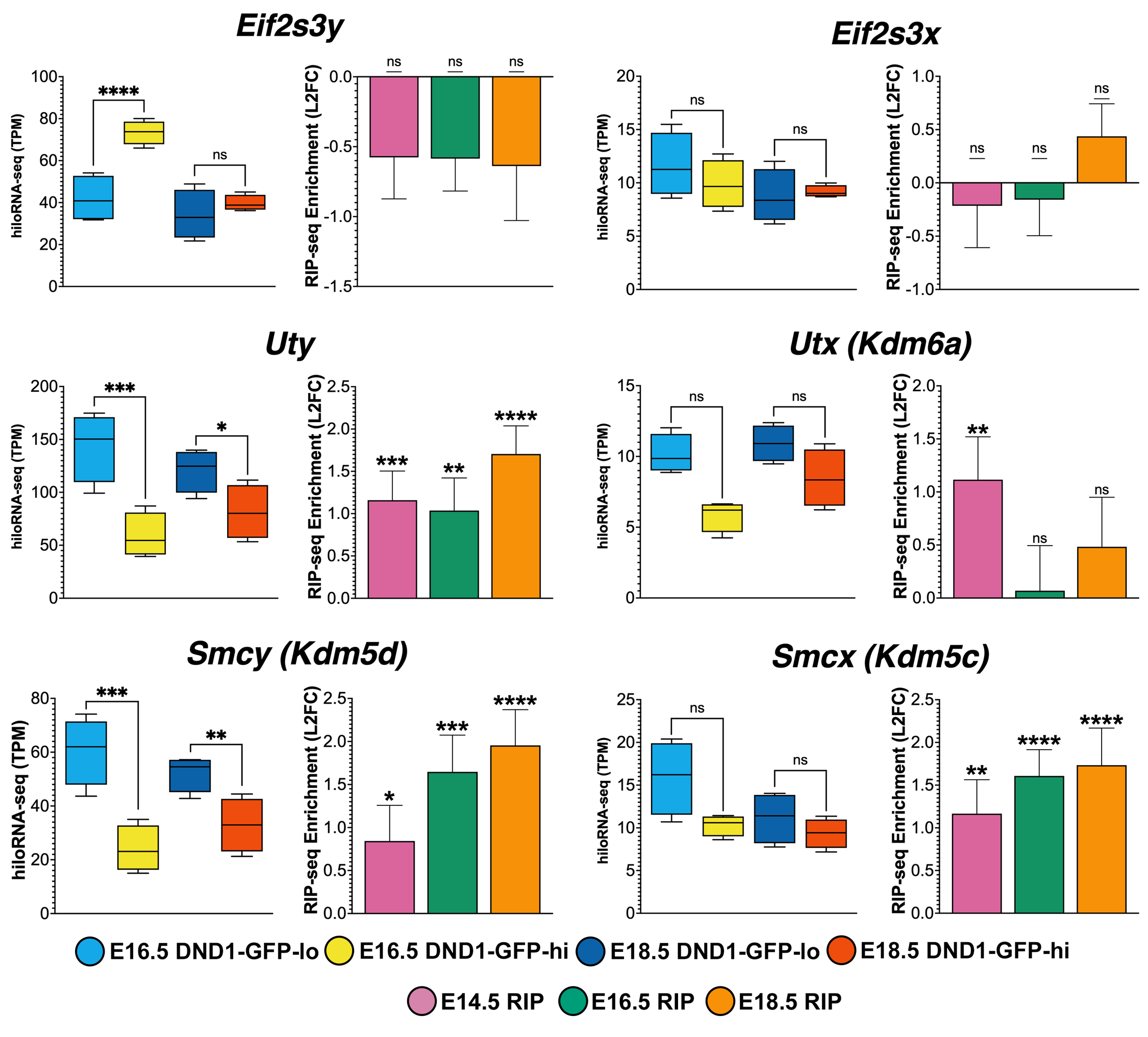

Supplement: S12 Fig — Gene-level hiloRNA-seq and RIP-seq data for Y-linked (Eif2s3y, Uty, Smcy) and X-linked (Eif2s3x, Utx, Smcx) genes. hiloRNA-seq expression P-value (DESeq2) between DND1-GFP-lo and DND1-GFP-hi cells at E16.5 and E18.5 and RIP-seq enrichment P-value (DESeq2): not significant (ns), <0.05 (*), <0.01 (**), <0.001 (***), <0.0001 (****). (TIF) [file pgen.1010656.s017.tif]

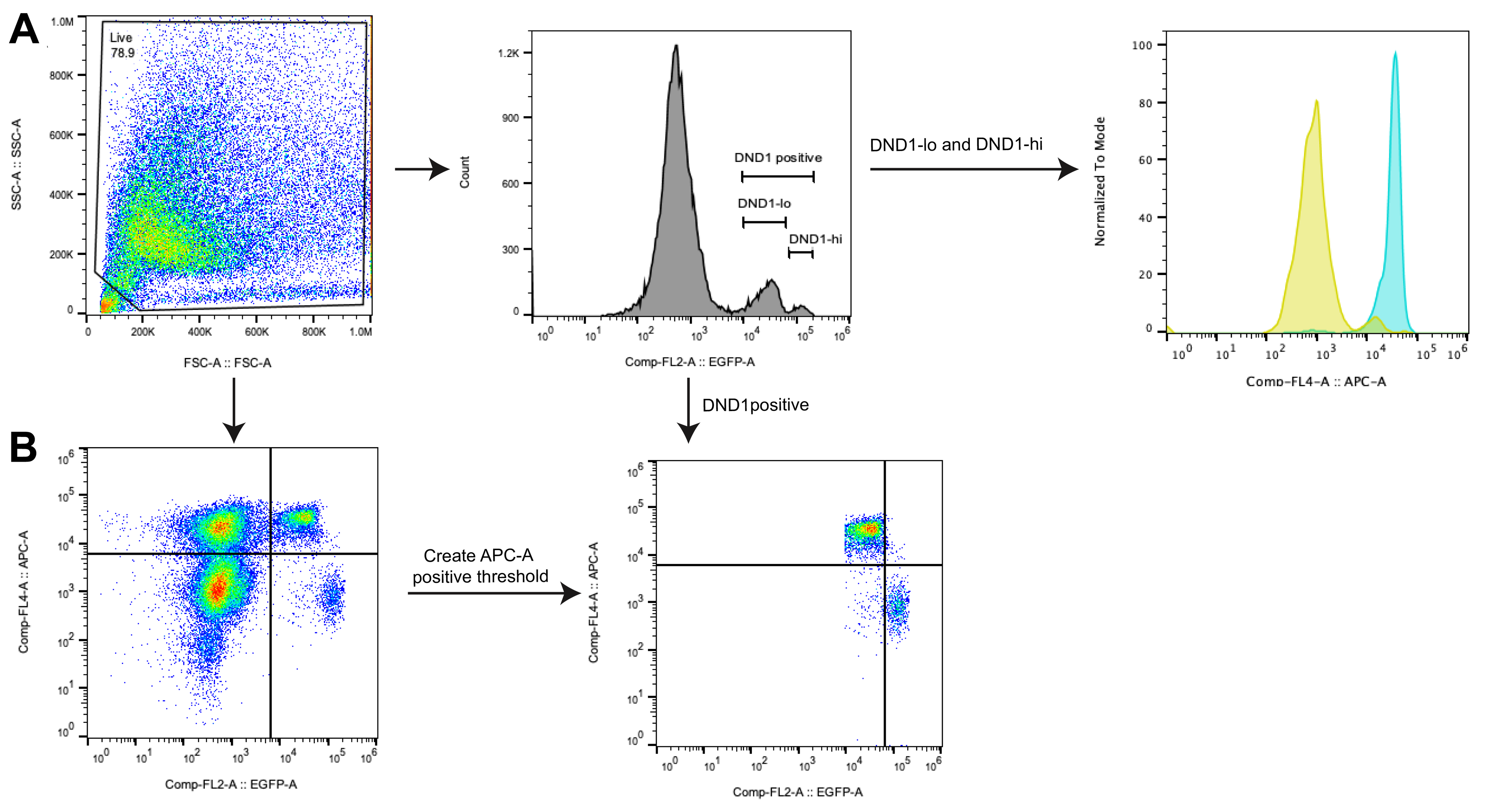

Supplement: S13 Fig — A: Live cells were gated using FSC-A and SSC-A parameters, and DND1-GFP-lo cells and DND1-GFP-hi cells were identified with GFP fluorescence and cell count parameters. DND1-GFP-lo and DND1-GFP-hi populations were plotted by 647/APC-A (Annexin) fluorescence. B: Annexin V-positive threshold was determined by examining live cells for GFP and 647/APC-A fluorescence. Using this threshold, the DND1-GFP positive cells were divided into DND1-GFP-hi and DND1-GFP-lo, creating four quadrants. (TIF) [file pgen.1010656.s018.tif]
