# Supplementary material for: The RNA binding protein DND1 is elevated in a subpopulation of pro-spermatogonia and targets chromatin modifiers and translational machinery during late gestation
Source: PLoS Genet. 2023 Mar 1;19(3):e1010656. doi: 10.1371/journal.pgen.1010656 (PMC10010562; doi:10.1371/journal.pgen.1010656)
Supplement: Attachment — Submitted filename: Ruthig_RTR.docx [file pgen.1010656.s019.docx]

Erez Raz
Guest Editor
PLOS Genetics

John Greally
Section Editor
PLOS Genetics


Dear Victor,
I am sending the referees comments on your paper.

Everyone agreed the work is of interest for readers of the Journal and that the data will serve as an important resource.
Two points were considered more important -

1. Provide clear criteria for defining cells as Hi or Lo when microscopy data is concerned (when co-staining with other markers). Was there a threshold above which cells were defined as "Hi"? Was there a control for the signal level at the same imaging plane?

We have developed a method for quantifying levels of DND1 relative to Hoechst and added these data to the manuscript.

2. A clear suggestion for a "take-home message" should be included. i.e., what is the functional significance of the Hi and Lo Dnd1-expressing populations for gamete formation?

Our new data showing that DND1-GFP-lo cells express high levels of phosphatidylserine on their outer plasma membrane (as detected by Annexin V) at E16.5, suggests that the DND1-GFP-hi cells are more likely to survive and give rise to pro-spermatogonia just after birth. We added these data to the manuscript and expanded on the functional significance in the Discussion.

We hope you find the comments of the referees useful!

Yes, in fact, the reviewers’ comments were very useful and made this a better paper.

Reviewer's Responses to Questions

**Comments to the Authors:
Please note here if the review is uploaded as an attachment.**

**Reviewer #1:** Overall
In this very well written manuscript, Ruthig et al identify populations of embryonic germ cells expressing either high or low levels of a Dnd1-GFP reporter knock-in allele. Using this allele, the authors identify fundamental transcriptional differences between these populations and direct RNA targets of the DND1 RNA binding protein. Several regulators of the epigenome and translational machinery are expressed at higher levels in Dnd1-hi cells and are shown to be direct targets of Dnd1. Overall, the experiments shown are very well thought out, performed appropriately, and data correctly interpreted. There are a couple of critical issues to address.

First, there is an early emphasis in the abstract and introduction on the role of Dnd1 in teratoma formation in mice based on experiments using the Dnd1-ter allele (with little mention of germ cell deficiency in all background or that teratomas are specific to one background). Although these hi and low cells might tell us something about teratoma formation experiments testing a functional connection are not performed and a teratoma susceptible background is not used. Teratomas are barely mentioned in the discussion. It is suggested that this early emphasis on teratomas be reduced or additional experiments be performed (likely outside the scope of the paper and not being requested).

We were unaware that we put so much emphasis on teratomas in the introduction. We certainly did not intend to do that, and we thank the reviewer for pointing this out, and have modified this section. We now mention the germ cell deficiency phenotype in mouse and zebrafish. Teratomas are mentioned in connection with the (somewhat) parallel finding in zebrafish that *dnd1* mutant germ cells undergo differentiation to somatic lineages. This suggests a loss of commitment to germ cell fate in mutants in both species. In *C. elegans*, germ cell fate is maintained by epigenetic mechanisms, which we feel connects to our findings that most epigenetic regulators are targets of DND1 and are higher in DND1-GFP-hi cells.

Second, and this addresses overall impact, it is still a bit unclear to me what the role of high Dnd1 during the time course of germ cell development is and where the Dnd1-hi population of cells sits in this process (from PGC to committed pro-spermatogonia). Is this a dynamic population, and representing the nexus at which differentiation is starting/completing? What is the critical nature of this event/stage of a germ cell? Why are pluripotency genes (e.g. Sox2) and critical male germ cell differentiation genes (e.g. Nanos2) expressed higher in these cells compared to Dnd1-low? I think the authors attempt to address this in the discussion but it does need some clarification as a hypothesis. Showing functional impact of high DND1 on germ cells (e.g. DNMT3L or SOX2 protein is detected more frequently or is higher in this population; cells are proliferating/not proliferating) would add significance if achievable.

We were also surprised that the level of the male differentiation gene, *Nanos*2, and the pluripotency genes (*Sox2, Tfap2c*, etc) were much higher in high cells. These cells also show high levels of genes associated with Oxidative Phosphorylation, and our new data show that the DND1-hi cells display much lower levels of phosphatidylserine on their surface, suggesting that they are more viable than DND1-GFP-lo cells, and consequently, we suggest DND1-hi are more likely to survive and give rise to pro-spermatogonia. Germ cells are not proliferating during this period from ~E14.5 until P2, so differences are not explained by (and do not affect) cell cycle. In future work, we intend to investigate the hypothesis that the epigenetic landscape is changed in DND1-hi cells.

Major comments
Abstract, introduction, and discussion: There is a lot of early emphasis on loss of Dnd1 and teratomas in mice. Couple of issues with this. First, the experiments conducted are not on a teratoma susceptible background and it isn’t mentioned anywhere that teratomas happen on a specific inbred background. Second, there is little said in the discussion tying the data to teratoma development. Third, the one mention of teratomas in the discussion is that de-regulation of epigenetic regulators in the absence of Dnd1 could lead to acquisition of somatic fate and teratomas. How does this work given that the first step in teratoma formation in mice is establishment of pluripotent embryonal carcinoma cells, in which somatic fate has yet to be established? I’d suggest de-emphasizing teratomas in the introduction and focusing on germ cell development/differentiation. Otherwise, data from a teratoma susceptible background – which is outside the scope of the paper (and data I am not asking for) – should be included.
Introduction, line 62-64 – again, teratoma forming capacity of E12.5 germ cells is limited to one strain. So differentiation pathways are “open” in this strain (using this data). Better data to show the general “openness” of these pathways in germ cells in general is the capacity to generate embryonic germ cell lines from early germ cells and that capacity being lost as sex-specific differentiation initiates.

We agree that the focus of this work is not on teratomas and have replaced most mentions of “teratomas” in the text and/or mentioned that teratomas arise only in some inbred strains of mice. As evidence for “open” embryonic pathways we have adopted the suggestion to replace formation of teratomas with the ability of MGCs at E12.5 to give rise to embryonic germ cell lines.

Introduction - Maybe discuss the (putative) molecular functions of Dnd1 up front. Doesn’t have to be a lot of detail (can leave for the discussion to integrate the findings of the paper into its known role), but something should be mentioned in the intro.

Lines 91-99 were added to provide functional information about DND1.

“Functionally, DND1 has been shown to stabilize some target transcripts by blocking microRNA-mediated destruction (24), and destabilize other target transcripts by recruitment to processing (P) bodies as part of the CNOT (carbon catabolite repressor 4-negative on TATA) complex (1, 2). Target transcript degradation via the CNOT complex relies on protein-protein interactions between DND1 and a second RNA binding protein NANOS2, which can modify DND1 target selection (1, 25). Recent structural analysis has shown that motif recognition prompts a two-step RNA binding mechanism resulting in oppositional binding by the two tandem RNA recognition motifs (RRM) of DND1 in a clamp fashion, while the double-stranded recognition motif (DSRM) remains exposed, putatively to be bound by a protein partner such as NANOS2 (26).”

Introduction – what experimental evidence is there that mouse germ cells “trans-differentiate” to form teratomas? I think that this is a point of contention in the field – are germ cells lineage specified prior to the time tumors form. Do they “escape” germ cell identity or fail to acquire/lock in identity. I’d suggest not using this term, provide specific evidence that they “trans-differentiate”, or indicate that the precise mechanism of germ cell transformation/trans-differentiation/de-dedifferentiation is not known.

Lines 56-63: We avoided the word “transform” and instead simply stated that MGCs differentiate to somatic fates.

“For example, in many inbred mouse strains, most germ cells carrying the Dnd1Ter/Ter mutation undergo apoptosis soon after specification (3, 4). On some genetic backgrounds, Dnd1Ter/Ter male germ cells (MGCs) that escape apoptosis often differentiate into teratomas with many embryonic cell types represented (3, 5). This is broadly consistent with findings in zebrafish, where some dnd1 mutant germ cells die, while others undergo differentiation to somatic lineages during migration to the gonad (6). These studies suggest that DND1 plays a critical role in maintaining germ cell identity by promoting survival and suppressing differentiation to somatic fate.”

Abstract, introduction, and discussion: The potential critical role of translational control/rates in teratoma formation from germ cells has been previously described (Heaney et al, Hum Mol Genetics, 2009). This work describes deficiency for Eif2s2 as reducing teratoma incidence in mice. Important considering that the authors address DND1 regulation of translation regulators/factors and specifically mention Eif2s3x and y). So, if teratomas are going to be part of the theme of the manuscript, it would be a good idea to reference this paper. These data are not in agreement with the concept of the role of Dnd1 in regulation of translation factors and teratomas. Dnd1-hi cells have higher expression of translation regulators (assuming higher rates of translation?) but should also maybe be more protected from forming teratomas? Dnd1 deficient cells should then maybe have less expression of these translation regulators but Dnd1-ter germ cells form embryonal carcinoma cells at a high rate in 129 mice.

We intend to address the role of DND1 in normal differentiation of MGCs, thus, we think the genetic interaction between Eif2s2 and DND1 in teratoma formation is less relevant to the current study. We are very interested in the idea that DND1’s interaction with Eif factors holds a key to understanding how DND1 promotes vs inhibits translation. This will be a topic of further study.

Results: Data in Fig S1 is very interesting. Is the GFP weaker in areas closest to the rete testis? Is there some biological significance to this? Or is this an imaging artifact?

We believe this is an imaging artifact due to the plane of section. We find DND1-GFP-hi and -lo cells scattered throughout the cords. We have enhanced these images with zooms on central and rete regions and added more images of native DND1-GFP signal in fixed cells that are more representative.

Results: Fig 1 - Does expression from the endogenous (unmodified) Dnd1 allele match the reporter? I’d assume that one could collect the hi and low cells and look at RNA expression in hets? Probably a better control to assess “transgene” artifact than going to independent RNA-seq studies. You have the hi and low populations separated here. Should be able to confirm native allele RNA expression. But, looks like dnd1 expression in the sorted populations might be embedded in the RNA-seq data (Fig 3C)? Might want to find a way of connecting these.

Based on the RNA-seq datasets, the hi or lo expression of DND1 is a property of the endogenous locus (e.g., it is not limited to the CRISPR-modified allele). However, we followed this suggestion and performed RT-PCR on DND1-hi and DND1-GFP-lo populations obtained from heterozygotes. Our data show that *Dnd*1 is expressed at similar levels from the endogenous and CRISPR-modified allele. These data are presented in Fig S2.

Results: Fig 2 – Using qualitative fluorescent intensity of the GFP reporter to look at whether hi and low GFP cells associate with these markers or not is probably not a great approach. I see the value of these data, but the RNa-seq data are much more convincing. I suggest moving to supporting info. There isnt much gained from these data.

We agree and moved former Fig. 2 to Supporting Information as Fig S5.

Results: Fig 3D (now Fig 2D) – not too sure what is being shown here. Is this a combination of different genes? If yes, this needs to be explained a bit better in the results (and which genes are involved).

We changed this section (lines 188-191) to read: “Gene ontology (GO) analysis on differentially expressed genes (DEGs) identified differences in energy metabolism between the DND1-GFP-hi and lo populations. Most striking of these metabolic differences was the increase of oxidative phosphorylation in DND1-GFP-hi cells (Fig 2D).”

Results: Line 200 – 202 “Despite the convergence of some transcripts, many epigenetic regulators maintain higher expression levels (TPM≥5.0, log2-fold change (L2FC)≥1.0, p-val<0.05) in DND1-GFP-hi cells during G0.” Can it really be said that these cells are in G0. I don’t think the proliferative status (or stage of cell cycle arrest – pH3 vs Ki67 staining) of the hi and low cells were assessed here. Is this known?

It is well established that MGCs enter G0 cell cycle arrest beginning at ~E14.5 and remain arrested until P2, when proliferation resumes. In lines 71-72, we have restated this with multiple references:

“During the last third of fetal life until a few days after birth (E14.5 to about P2), MGCs enter a long period of cell cycle arrest in G0 (14-16).”

Nagano et al. 2000 The Anatomical Record

Western et al. 2008 Stem Cells

Cook et al. 2011 Development

Shimada et al. 2021 iScience

Zhao et al. 2021 Nature Communications

Results and discussion: After reading the discussion, I was still left with the questions – what is the fundamental difference between Ddn1-hi and low? Other than differences in gene expression – how are they functionally different. Proliferating/not proliferating? Still expressing pluripotency factors (protein)/not retain pluripotent characteristics? Initiated NANOS2 or DNMT3 (a or l) protein expression or not? For example, Dnmt3a and Dnmt3l RNA are expressed higher in DND1-hi cells. How does this translate to protein? Are the proteins differentially present/absent in the DND1-low and hi populations? Maybe can do this by imaging? Are the proteins expressed differently? Understand that this is much harder as westerns would be terribly difficult with cell numbers available.

We agree that examining protein expression between the two populations is important, and while we would like to follow this line of investigation. As the reviewer points out, Westerns would be very difficult and antibody availability for IF is currently very limiting. We tried staining with an antibody against DNMT3A, but results were inconclusive. We have initiated experiments to perform proteomic analysis. We are hopeful that this will be informative, but feel this work is beyond the scope of this paper. To begin to address the functional difference between the two populations, we examined the presence of phosphatidylserine on the plasma membrane, a signal for phagocytosis, in E16.5 DND1-hi and DND1-lo cells through Annexin V detection. We see that DND1-lo cells exhibit significantly higher Annexin V fluorescence, as compared to DND1-hi cells and found that *Anxa5*, the gene encoding the Annexin V transcript, is a strong DND1 target throughout our RIP-seq time course. *Anxa5* is also significantly over-expressed in E16.5 DND1-GFP-lo cells and appears in multiple Gene Ontology lists related to apoptosis. These results suggest that DND1-hi cells are more likely to survive and give rise to pro-spermatogonia. These data have been added to the manuscript and provide evidence for a functional difference of DND1-GFP-hi and DND1-GFP-lo populations that will be further explored in future studies.

Results: Most of the direct targets of DND1 appear to be expressed higher in DND1-hi cells. Are there examples of transcripts that are direct targets of DND1 and are lower in DND1-hi cells? Gets to an overall.

Yes, although more DND1 targets are expressed at higher levels in DND1-GFP-hi cells, there are some targets that are expressed at higher levels in DND1-GFP-lo cells. Of the 869 DND1 transcript targets identified in our analysis, 164 were found to be higher in E16.5 DND1-hi cells (18.87%), 81 in E18.5 DND1-hi cells (9.32%), 81 in E16.5 DND1-lo cells (9.32%), and 6 in E18.5 DND1-lo cells (0.01%). These details were added in lines 335-337.

Discussion: Line 341 – 344. Nanos2 is not a marker of pluripotency. In fact, having some genes such as Sox2 (a marker of pluripotency) and a male germ regulator of sex-specific differentiation (Nanos2) both increased in expression in Dnd1-hi cells is counterintuitive. This should be addressed in the discussion. Other genes that are indicative of male germ cell differentiation (Tdrd’s, methyltransferases) are also higher. So why would Sox2, Tfap2c be higher? Are these cells that are being actively transitioned from a more “PGC-like” state to a more “differentiated” state so we are seeing this in the transcriptional profiles? Maybe I missed it, but is Sox2 a target of Dnd1? If not, would support that these Dnd1-hi cells are being actively pushed to differentiate by high Dnd1?

We agree that these cells are in transition, still retaining some pluripotent characteristics, but also expressing male differentiation genes. We have altered the text to state this more clearly in lines 427-436.

“Consistent with the idea that DND1-GFP-hi cells are a select population of MGCs, at E16.5, they express much higher levels of genes associated with associated with the germline stem cell population including Dazl, Ddx4, Nanos2, Dppa3, Tfap2c, Pou5f1, and Sox2, although transcripts for all these genes decline by E18.5. DND1-GFP-hi cells show higher expression of genes associated with oxidative phosphorylation, and also express elevated levels of other RNA binding proteins relevant to male fate specification such as Dazl and Nanos2. DAZL is associated with the transition to MGC differentiation (63) and NANOS2 is well documented to work in complex with DND1 to target transcripts of pluripotency genes and activating cyclins for degradation (1, 25, 64). Dazl has also been shown to be targeted by NANOS2 as part of negative regulation and commitment to MGC fate (65)”

Minor comments

Overall, the figures are put together very well. Recommend that the text sizing be adjusted for some panels (e.g. 3C, 7B) as they are very hard to read without zooming in A LOT.

We have increased the text sizing as suggested.

Results: Probably best to refer to the allele as a knock-in, not a transgene which usually is used when describing a randomly integrated DNA construct.

We have made this change.

**Reviewer #2**: The manuscript by Ruthig et al., describes molecular phenotyping analysis of a subpopulation of male germ cells in mouse, which has been identified by transgenic insertion of GFP into DND1 gene and detection of a selectively high DND1 expressing cell population. The discovery of this cell population by the transgene is validated by endogenous DND1 expression analysis in single cell transcriptome and further analysed by differential gene expression analysis. This analysis suggest striking heterogeneity and associated differences in gene expression profiles between the two cell populations. Differentially expressed genes suggest DND1-associated regulation of cell cycle, cellular metabolism, protein translation and chromatin state regulator genes. Of note, several of these functions were already suggested before in the literature. Subsequently, the authors identify RNAs bound by DND1 using RIP and suggest that the two datasets indicate roles for DND1 in regulating subsets of RNAs and affect translation of target genes. The authors main claim is that the different DND1 levels indicate male germ cell heterogeneity, and their results pinpoint features of the heterogenous cell populatjons using their transcriptomic and RIP analyses. The manuscript is primarily descriptive but may represent potentially important, biologically relevant description of germ cell heterogeneity and may contribute to better understanding of the role of DND1 in germ cell fate if the heterogeneity detected is better linked to more comprehensive cell biological/histological characterisation of the novel cell population and if the transcriptome and RIP results are more robustly correlated.

There are several questions arising from the current version of the ms, which need addressing (particularly Q1-3).

1. Does the heterogeneity detected by the transgenic insertion of GFP marker faithfully represent true endogenous (non-transgenic) heterogeneity? While single cell data suggest that high expressing DND1 cells indeed exist, are those cells in the single cell data showing similar transcript dynamic of candidate targets and DEGs seen in the FACS isolated cells?

We liked this idea and assessed the expression levels of a group of epigenetic and pluripotency regulation and differentiation genes expressed at higher levels in DND1-GFP-hi cells (*Brdt, Dnmt3a, Mael, Piwil4, Dazl, Dppa5a*) and DND1-GFP-lo cells *Kdm6b, Lefty2*) in the Oatley dataset. Fig S4 now shows that all 8 of these genes are also more highly expressed in wild-type cells expressing higher levels of *Dnd1*.

2. What is the cellular and functional relevance of the molecular heterogeneity detected in this ms? This question is important for assessing the meaning of the detected transcriptomic differences, given that the authors do not see correlation between the high DND1 expression and known variable features of germ cells such as differentiating or apoptotic germ cells. Without providing some validation to the observed DEG and RNA targeting phenomena the study will not represent sufficient advance. A complementing histological characterization of the high DND1 cells will also offer the benefits of validation of the predicted molecular (e.g. chromatin remodeling) and cellular (e.g. cell cycle) functions highlighted by the authors. Such characterization/validation may include high DND1 cell group-specific detection of changes in cell cycle markers, detection of expected global changes in repressive chromatin states (e.g. comparative immunohistochemistry of DNA and histone methylation markers in high and low DND1 cells), analysis of global translation regulation and oxidative phosphorylation.

Our new data, showing that the DND1-GFP-lo cells display much higher levels of phosphatidylserine on their surface (as detected by Annexin V), suggest that the DND1-GFP-hi cells are more likely to survive and give rise to the pro-spermatogonial population. However, we do not know the basis for this selection, and we do not know whether cells can cycle in and out of a DND1-high state. We are currently trying to address these questions, but for the moment, we want to be careful about our conclusions. Since we hypothesize that DND1 regulates epigenetic enzymes, we plan to investigate epigenetic differences between DND1-GFP-hi and DND1-GFP-lo cells in future work.

3. A related question to that above, is whether the authors see differential subcellular localisation patterns of DND1 and or cell distributions at different stages of germ cell development between DND1 high and low cells? Is there any change in cellular behaviour that may indicate the distinct fates between these cell groups? Figure 3 indicates some clustering of DND1 high cells. Is this stereotypical for tissue position or frequency of clustering?

We have performed quantification of DND1 levels in individual cells and added this to the manuscript. In the course of this work, we did not see cellular localization patterns that were consistently different between DND1-GFP-hi and DND1-GFP-lo cells. DND1 is in the cytoplasm, the nucleus and the processing (P) bodies in both populations. We often do see some clustering of DND1-GFP-hi and DND1-GFP-lo cells inside cords, but their localization is not stereotypic within the testis.

4. It is difficult to reconcile or appreciate the partial overlap between DEG and RIP results. The composition of the subsets of genes that are differentially expressed in high DND1 cells that appear to be direct targets could be explained by the composite effect of DND1 dependent stabilization/degradation of RNAs and the indirect effect of DND1-dependent translation regulation associated RNA degradation. One would expect tighter correlation between temporal expression dynamic (DEG) and temporal degree of DND1 targeting (RIP) at the different stages of germ cell development tested upon direct effect. Can such temporal analysis of correlation between the expression changes and RIP results in E16.5-E18.5 be carried out?

We agree this is confusing. Although more DND1 targets are expressed at higher levels in DND1-GFP-hi cells, there are some targets that are expressed at higher levels in DND1-GFP-lo cells. This could be because these are targets DND1 degrades (so they are under-represented in DND1-GFP-hi cells), but there are other possibilities. Of the 869 DND1 transcript targets identified in our analysis, 164 were found in E16.5 DND1-GFP-hi cells (18.87%), 81 in E18.5 DND1-GFP-hi cells (9.32%), 81 in E16.5 DND1-GFP-lo cells (9.32%), and 6 in E18.5 DND1-GFP-lo cells (0.01%). These details were added in lines 335-337. Because DND1 can protect or degrade targets, we think we can only untangle this problem when we have proteomic data.

5. Not essential but certainly relevant question and relatively easy to check whether the DND1 targets match those which are dependent on DND1 homologs in other organisms (e.g. zebrafish).

We have included a short comment on this in the discussion:

“While other targets identified by us (*Tdrd7*, *Rock2*, *Mylk*, *Anxa5*) have previously been reported as targets of DND in zebrafish Goudarzi et al., 2012; Kedden et al., 2007).” Line 408-409

Minor points:
1. The data in Figure 4 is hard to follow due to the multiple colours which are difficult to assign using the colour code in the legends. Can the authors revise this figure to link the DEG categories shown in stack bars across stages and show the GO terms next to the stacks?

We have reformatted this figure and hope that it is easier to digest.

2. Font size in Fig. 7B should be increased.

We have increased this font size.

3. Protein is misspelt in Figure 4 legend.

Thanks for catching this – fixed.

4. There is no need to abbreviate the word Figure in Figure titles (or indeed throughout the ms).

We have used the convention required by *PLoS Genetics*.

**Reviewer #3**: Ruthig et al report on two populations of male germ cells characterized by different levels of Dead end expression. The difference between the two population is detected by the expression level of a fusion between DND1 and GFP that is inserted within the endogenous locus. The cell population expressing higher level of DND1-GFP show higher level of epigenetic regulators RNAs, and the protein binds RNAs of such regulators.
As DND plays central roles in the development of germ cells, the lists of possible DND1 in vivo targets are important resource for future functional studies. The results are thus clearly of interest for the readership of the Journal.

Addressing/explaining better some of the issues listed below would improve the manuscript.

Introduction

1. “During the last third of fetal life until a few days after birth (E14.5 to about P2) (7-9), male germ cells (MGCs) enter a long period of cell cycle arrest, during which they undergo extensive reprogramming that reduces their potential to give rise to teratomas and drives cells towards the pro-spermatogonial (PSG) fate.” – has it been critically shown that “extensive reprogramming” at these stages indeed reduces teratoma formation? If so, the source should be cited. In certain contexts, reprogramming is thought to promote teratoma formation, so would be good if the authors define “reprogramming” in their case and align them with previous literature (e.g. <https://www.nature.com/articles/nature12586> ).

We appreciate the reviewer’s point and thank him/her for directing us to *Abad et al. 2013 Nature*. Although there is plenty of evidence that the potential of MGCs to give rise to teratomas or embryonic germ cell lines declines rapidly during the period of cell cycle arrest (Stevens 1958 J Natl Canc Inst, Matsui et al. 1992, Cell, Kimura et al. 2008 Dev), and that epigenetic changes also occur during this window of development (Seisenberger et al. 2012 Mol Cell, Lucifero et al. 2002 Genomics, Harvan-Calvopina et al. 2016 Dev Cell, Oliveros-Etter et al. 2015 Stem Cells Rep Clark), there is no direct evidence that epigenetic reprogramming is causal to the decline in “pluripotency”. Most of the literature around stem cell differentiation would support this idea, but we have rewritten this section in lines 71-74 with the reviewer’s suggestions in mind.

Leroy Stevens initially demonstrated in 1958 (J Natl Canc Inst) that transplanting PGCs after E13.5 produces teratomas with very low efficiency. Matsui et al. showed in 1992 (Cell) that the ability to derive EGCs from PGCs declines by E13.5. Kimura et al. demonstrated in 2008 (Development) that EGCs cannot be derived from PGCs after E15.5. We have also mentioned a group of papers that investigated the unique way male germ cells regulate pluripotency in the context of cell cycle arrest, and the impacts of a derailed differentiation program (Heaney et al. 2012 Dev, Dawson et al. 2018 Dev, Western et al. 2010 FASEB J)

- Stevens, L. C. (1958). "Studies on transplantable testicular teratomas of strain 129 mice." J Natl Cancer Inst 20(6): 1257-1275.
- Matsui, Y., K. Zsebo and B. L. Hogan (1992). "Derivation of pluripotential embryonic stem cells from murine primordial germ cells in culture." Cell 70(5): 841-847.
- Kimura, T., M. Tomooka, N. Yamano, K. Murayama, S. Matoba, H. Umehara, Y. Kanai and T. Nakano (2008). "AKT signaling promotes derivation of embryonic germ cells from primordial germ cells." Development 135(5): 869-879.
- Seisenberger, S., S. Andrews, F. Krueger, J. Arand, J. Walter, F. Santos, C. Popp, B. Thienpont, W. Dean and W. Reik (2012). "The dynamics of genome-wide DNA methylation reprogramming in mouse primordial germ cells." Molecular Cell 48(6): 849-862.
- Lucifero, D., C. Mertineit, H. J. Clarke, T. H. Bestor and J. M. Trasler (2002). "Methylation dynamics of imprinted genes in mouse germ cells." Genomics 79(4): 530-538.
- Hargan-Calvopina, J., S. Taylor, H. Cook, Z. Hu, S. A. Lee, M. R. Yen, Y. S. Chiang, P. Y. Chen and A. T. Clark (2016). "Stage-Specific Demethylation in Primordial Germ Cells Safeguards against Precocious Differentiation." Dev Cell 39(1): 75-86.
- Oliveros-Etter, M., Z. Li, K. Nee, L. Hosohama, J. Hargan-Calvopina, S. A. Lee, P. Joti, J. Yu and A. T. Clark (2015). "PGC Reversion to Pluripotency Involves Erasure of DNA Methylation from Imprinting Control Centers followed by Locus-Specific Re-methylation." Stem Cell Reports 5(3): 337-349.
- Heaney, J. D., E. L. Anderson, M. V. Michelson, J. L. Zechel, P. A. Conrad, D. C. Page and J. H. Nadeau (2012). "Germ cell pluripotency, premature differentiation and susceptibility to testicular teratomas in mice." Development 139(9): 1577-1586.
- Dawson, E. P., D. G. Lanza, N. J. Webster, S. M. Benton, I. Suetake and J. D. Heaney (2018). "Delayed male germ cell sex-specification permits transition into embryonal carcinoma cells with features of primed pluripotency." Development 145(6).
- Western, P. S., J. A. van den Bergen, D. C. Miles and A. H. Sinclair (2010). "Male fetal germ cell differentiation involves complex repression of the regulatory network controlling pluripotency." FASEB J 24(8): 3026-3035.

2. “Dead end 1 (Dnd1), an RNA binding protein (RBP), underlies the classic mouse mutation “Ter”, named for the high incidence of teratomas in male Dnd1Ter/Ter mutants” – mention that this holds true for a specific genetic background only.

We have adopted this suggestion (line 58-60).

3. “loss or mutation of Dnd1 leads to a severe loss of embryonic germ cells soon after their specification (17, 18), and in zebrafish, transformation of germ cells to somatic lineages” – not clear what “severe loss” means. Would “loss“ be sufficient. In addition, the way it is phrased, it sounds as if the apparent loss of cells in the mouse is not (also) a result of cell transformation. Is cell death the only way of losing the germ cells in Ter mice?

We modified this section in lines 55-58. Over a period of 3-4 years, we tried to lineage trace MGCs carrying the Ter mutation in mouse, but we were not successful. Ter mice are very poor breeders and we never succeeded in getting a mouse carrying Oct4-Cre; RTM; Ter/Ter. Though we suspect we would see the same thing that Raz found in zebrafish, but we gave up carrying the line. We are all still disappointed about this!

4. “Although some of these chromatin modifiers mapped as mRNA targets of DND1 in HEK293 cells, many were not expressed in this cell line” – This sentence is clear for those who read reference 21. I would add 2 sentences, explaining the results presented in references 20 and 21 such that this part of the introduction is a “stand alone” paragraph.

We thank the reviewer for pointing this out. We modified this section to add information from these two references in lines 85-91.

Results

5. “At E14.5, the DND1-GFP-hi population accounts for <1% of MGCs. Their numbers peak at E16.5, when DND1-GFP-hi cells represent 12% of total MGCs, and decline again at E18.5, when the DND1-GFP-hi population accounts for only 5% of MGCs. “. In table S2, the numbers and percentages do not add up properly (are not always 100%, the total MGCs is less/more than hi + lo). Is there an explanation for that?

This was a mistake in the original calculations. The number of lo cells is now derived from the difference between the total number of cells and the number of hi cells so that the percentages then add up to exactly 100% at each timepoint. We thank the reviewer for catching this and have made the corrections.

6. What stage is presented in Fig 1A?

We adjusted this legend to say that this histogram is from E18.5.

7. In S1 E16, what should one consider as hi and lo ? there are regions that appear to be devoid of signal, some lo and some hi. The definition, of hi and lo should be made clear here. As presented, it seems like there are domains of hi on either side of the cords and that they are larger than what one would have expect from a 12% population size.

We have added insets to Fig S1 to help with this problem. Although the proportion of DND1-GFP-hi cells seems higher in IF, it is reproducibly lower in FACS analysis. We have also been concerned about this point and devised a method for quantifying expression of DND1-GFP in individual cells (Fig S2). The low proportion of DND1-GFP-hi cells was also found using our quantification protocol in which we score levels of DND1 normalized to Hoechst.

8. Define GCNA in Fig 2. What does the White “DNA” in 2A-C stand for? Why not show the level of these specific markers derived from the RNA seq done at E16?

This figure was moved to Supporting Information (Fig S5), and the legend was amended as follows:

“All sections stained for DND1-GFP (GFP, green), germ cell nuclear antigen (GCNA, blue), and DNA (Hoechst, white).”

We followed up this suggestion. L1td1 is slightly higher in DND1-GFP-lo cells, and Tomm20 is slightly higher in DND1-GFP-hi cells, especially at E18.5, but neither shows significant differences (consistent with the IF results). Although Parp1 is higher in hi cells, cleaved PARP (c-PARP) is not necessarily correlated with mRNA levels for Parp1. We do not think that the addition of this mRNA data to the manuscript would be valuable.

9. The authors should try to exclude the option that the hi and lo are not a result of small differences in the rate of development among cells at E16.5-E18.5, with all the cells becoming similar to each other at P3 (perhaps hi). It is not obvious how can one compare the lo in P3 with that in E18.5. If it is based on absolute GFP level it could signify more uniform distribution of cell states at the E14.5 and P3 -> It would help if the authors present arguments that make this option (that the small hi population is not simply composed of “faster”, or “slower” cells) less likely.

We do not think we can formally exclude the possibility that there are differences in the rate of development between DND1-GFP-hi cells and DND1-GFP-lo cells—in fact, differences in developmental rate could be due to DND1 levels. We think the additional data showing that binding of Annexin V is higher in DND1-GFP-lo cells suggest that there are functional/viability differences between these two populations. Interpretations of DND1 levels after P2 are complicated by the fact that some DND1+ MGCs reenter cell cycle and begin differentiation as spermatogonia, some enter meiosis, and others take up residence in the SSC compartment. During cell cycle arrest, heterogeneous levels of DND1 are surprising, but at P3, differences in DND1 levels are associated with the heterogeneous fates that emerge.

10. Conducting analysis similar to that presented in Fig 2 for 1-2 differentially-expressed markers derived from Fig 3 (antibody as in 2, or employing a quantitative in situ hybridization method) would increase the confidence in the results presented in both figures – i.e., show the correlations shown in 3 following the experimental scheme of Figure 2.

Conducting quantitative in situ hybridization of select transcripts and to understand how these transcripts are processed in DND1-GFP-hi and DND1-GFP-lo cells are the next steps in this project. As such, we agree with the reviewer that the in situ hybridization of transcripts would increase the confidence of our RNAseq results. However, we believe that our analysis of differentially-expressed genes in DND1-GFP-hi and DND1-GFP-lo populations from independent single cell sequencing datasets (please refer to Fig S4) sufficiently validates our RNAseq results within the scope of this study.

11. What justifies presenting the hi and lo of different stages within the same graphs in Figure 6? Were the counts normalized to the expression level of a house-keeping gene for example?

We are justified comparing expression data from DND1-GFP-hi and DND1-GFP-lo cells at different stages because Salmon was used to quantify all of the RNA-seq data, both RIP-seq and hiloRNA-seq, which produces expression values in TPM (Transcripts Per Kilobase Milllion). TPM calculations normalize first for gene length (per kilobase) and second for sequencing depth (scaling factor of 1,000,000 aka per million). This order of operations means that the total TPM for all genes of different samples is the same, because they are all proportional to the same scaling factor, and thus comparable to one another between samples. Quantification in this case was all done against the same index and with the same version of Salmon. The ability for equitable comparison can erode when comparing different tissue types, or when there is high variability in mRNA between samples. In our case we were only concerned with germ cells from testis tissue and our initial step before sequencing was to do quality control on mRNA levels to ensure submitted samples for sequencing were above a quality threshold.

12. The ideal control for the RIP would have been a GFP-tagged mutated Dnd1, but such a knockin is obviously not available. A control of RIP using another GFP fusion could have helped here. Perhaps I missed it, but what would increase the confidence in the RIP results and show specificity would be a demonstration of lack of very good (not just a few examples provided) correlation between the normalized expression level of transcripts (not rRNAs which are not polyadenylated) and the RIP results. The authors should just present the results more clearly to exclude this option.

Below we show examples of several genes (*Gapdh* and *Acta2*) that are highly expressed at each stage (input) but show low representation in the RIP results at each stage, as compared to a gene that shows high enrichment in the DND1 RIP data (*Lamb1*). We explain our approach in lines 286-288: “We used differential analysis between paired RNA input and immunoprecipitation (IP) samples to determine significant enrichment / non-enrichment of transcript binding targets of DND1 (Fig S9B; Table S4)”

We have added these examples to Figure S9B as a second step in data validation.

13. “ Nearly all of these chromatin regulators are genes that fail to be up-regulated in DND1Ter/Ter mutant germ cells by E14.5 “. Here the authors focus on RNAs encoding for proteins that are more “interesting” in the context of this work. How do other genes behave in this comparison?

Of course, there are many categories of genes that could be investigated, but we felt it was important to focus the manuscript on ones we felt make the most sense at present. We hope that others will use this data to investigate other aspects of MGC differentiation. That said, we also did triple dataset filtering for other GO terms including “Extracellular Matrix Organization”, “Collagen Family Members” and “Cell Cycle”. None of these showed consistent patterns. This data is shown in Table S3 and Figure S10A. We’ve mentioned this in Lines 354-359 and Lines 364-367. Note that the DND1^Ter/Ter^ MGC dataset encompasses stages E12.5, E13.5 and E14.5, so has limited usefulness for the 3-way comparison since only the first timepoint is overlapping.

Discussion

14. Is there anything special in germ cells at the stages studied that could account for the high representation of vesicle transport encoding RNAs?

We agree this is a very interesting question: we hope we will find an answer in the future.

**Have all data underlying the figures and results presented in the manuscript been provided?**
Large-scale datasets should be made available via a public repository as described in the *PLOS Genetics* [data availability policy](http://journals.plos.org/plosgenetics/s/data-availability), and numerical data that underlies graphs or summary statistics should be provided in spreadsheet form as supporting information.

Reviewer #1: Yes

Reviewer #2: Yes

Reviewer #3: Yes

PLOS authors have the option to publish the peer review history of their article ([what does this mean?](https://journals.plos.org/plosgenetics/s/editorial-and-peer-review-process#loc-peer-review-history)). If published, this will include your full peer review and any attached files.


If you choose “no”, your identity will remain anonymous but your review may still be made public.

**Do you want your identity to be public for this peer review?** For information about this choice, including consent withdrawal, please see our [Privacy Policy](https://www.plos.org/privacy-policy).

Reviewer #1: No

Reviewer #2: No

Reviewer #3: No
